# Inter and intra-observer agreement of arterial wall contrast-enhanced ultrasonography in giant cell arteritis

**DOI:** 10.3389/fmed.2022.1042366

**Published:** 2022-11-17

**Authors:** Olivier Espitia, Olivier Robin, Jeanne Hersant, Christophe Roncato, Arthur Théry, Marie-Anne Vibet, Giovanni Gautier, Alizée Raimbeau, François-Xavier Lapébie

**Affiliations:** ^1^Nantes Université, Centre Hospitalier Universitaire (CHU) Nantes, Department of Internal and Vascular Medicine, Nantes, France; ^2^Department of Vascular Medicine, Centre Hospitalier Universitaire (CHU) Angers, Angers, France; ^3^Department of Vascular Medicine, Centre Hospitalier Saint-Louis, La Rochelle, France; ^4^Department of Vascular Medicine, Centre Hospitalier Universitaire (CHU) Toulouse, Toulouse, France; ^5^Department of Biostatistics, Centre Hospitalier Universitaire (CHU) Nantes, Nantes, France

**Keywords:** contrast-enhanced ultrasonography (CEUS), giant cell arteritis–large-vessel, agreement, giant cell arteritis, large-vessel vasculitis (LVV)

## Abstract

**Objective:**

The aim of this study was to analyze inter- and intra-observer agreement for contrast-enhanced ultrasonography (CEUS) for monitoring disease activity in Giant Cell Arteritis (GCA) in the wall of axillary arteries, and common carotid arteries.

**Methods:**

Giant cell arteritis patients have CEUS of axillary arteries and common carotid. These images were rated by seven vascular medicine physicians from four hospitals who were experienced in duplex ultrasonography of GCA patients. Two weeks later, observers again rated the same images. GCA patients were recruited in from December 2019 to February 2021. An analysis of the contrast of the ultrasound images with a gradation in three classes (grade 0, 1, and 2) was performed. Grade 0 corresponds to no contrast, grade 1 to moderate wall contrast and grade 2 to intense contrast. A new analysis in 2 classes: positive or negative wall contrast; was then performed on new series of images.

**Results:**

Sixty arterial segments were evaluated in 30 patients. For the three-class scale, intra-rater agreement was substantial: κ 0.70; inter-rater agreement was fair: κ from 0.22 to 0.27. Thirty-four videos had a wall thickness of less than 2 mm and 26 videos had a wall thickness greater than 2 mm. For walls with a thickness lower than 2 mm: intra-rater agreement was substantial: κ 0.69; inter-rater agreement was fair: κ 0.35. For walls with a thickness of 2 mm or more: intra-rater agreement was substantial: κ 0.53; inter-rater agreement was fair: κ 0.25. For analysis of parietal contrast uptake in two classes: inter-rater agreement was fair to moderate: κ from 0.35 to 0.41; and for walls with a thickness of 2 mm or more: inter-rater agreement was fair to substantial κ from 0.22 to 0.63.

**Conclusion:**

The visual analysis of contrast uptake in the wall of the axillary and common carotid arteries showed good intra-rater agreement in GCA patients. The inter-rater agreement was low, especially when contrast was analyzed in three classes. The inter-rater agreement for the analysis in two classes was also low. The inter-rater agreement was higher in two-class analysis for walls of 2 mm thickness or more.

## Introduction

Giant cell arteritis (GCA) is the most common vasculitis in elderly people, with large-vessel vasculitis (LVV) involvement in slightly more than half of the GCA cases, such as the aorta and its branches particularly the axillary artery (1–3). GCA is characterized by an arterial wall inflammatory process within the vessel wall leading to structural arterial wall alterations from mild thickening until arterial occlusion, with late complications as aneurysm (4). Assessment of arterial wall inflammatory activity is important for monitoring GCA activity. Traditionally, the GCA evaluation was based on the clinical signs with monitoring of biological inflammatory markers such as erythrocyte sedimentation rate or C-reactive protein (CRP), but with the use of interleukin-6 receptor blockers, these biological parameters are becoming less informative. More recently, imaging by computed tomography (CT) scan, positron emission tomography (PET/CT) scan, color Doppler ultrasonography (CDUS) or magnetic resonance imaging (MRI), have become very important in the diagnosis of GCA but their use for the follow-up, in particular to evaluate the LVV activity of the disease, remains to be specified. Follow-up imaging data are heterogeneous, mainly because of a lack of standardization in the interpretation of these images.

Contrast-enhanced ultrasound (CEUS) was developed for a better vascular visualization. This examination is an ultrasound in B-mode, associated with an injection of ultrasound contrast. It consists of microbubbles of weakly soluble sulfur hexafluoride gas stabilized by a phospholipid and palmitic acid envelope, which allows an increase in circulation time after intravenous injection and therefore an increase in the duration of the examination (5). These microbubbles remain strictly localized to the vascular compartment. They are eliminated within 15 min after the injection.

Contrast-enhanced ultrasound was developed to improve the visualization of the vessel lumen and to identify unstable carotid plaques at an increased risk of stroke. These unstable plaques are characterized by the presence of intraplaque inflammation, leading to the formation of neovascularization that are likely to rupture, which may result in plaque fissure, thrombus formation, and stroke (5, 6). Injection of an ultrasound contrast medium allows ultrasound visualization of microbubbles circulating in these neo-vessels. CEUS is also used to improve vascular visualization in aortic prosthesis monitoring and in digestive vascular imaging.

In GCA, arteries could be evaluated using B-mode and CDUS imaging. Typical signs of GCA are circumferential, homogeneous, hypo-echogenic wall thickening (“halo sign”) or compression sign for temporal arteritis (7–9). The intima-media thickness (IMT) ≥1 mm cutoff value, in the axillary artery has sensitivity and specificity values of 96.1–100% for GCA but 6 months after GCA treatment, approximately 50% of the patients had persistent arterial thickening despite normalization of biological inflammatory markers and the absence of clinical symptoms (10–12). To improve wall thickening analysis, CEUS could be used for vascular imaging, especially for patients with a persistent thickened vessel wall in large-vessels.

Studies using CEUS in patients with GCA or Takayasu arteritis (TA) describe uptake of ultrasound contrast agent into the vessel wall in active vasculitis (11–16). Previously published studies with CEUS in LVV used a semi-quantitative score with three-class scale (6). CEUS could detect an increase in the vascularization of the wall of these arteries, which seems to correlate with the activity (11, 17, 18). Few studies have been performed in GCA patients and they most often report GCA patients associated with TA patients. A pilot study of seven patients with TA (*n* = 5) or GCA (*n* = 2) has evaluated CDUS and CEUS of the carotid arteries (14). Of the 14 carotid arteries examined, 50% had lesions on CDUS (parietal thickening), and 64% had neovascularization of the wall on CEUS. CEUS was positive on both carotid arteries in one patient while the CDUS was negative, and conversely, parietal thickening was noted on one carotid artery in one patient on CDUS without contrast uptake on CEUS. Another study compared CEUS and PET/CT of the carotid arteries in a series of 31 consecutive patients with TA (*n* = 14), or GCA with LLV on PET/CT (*n* = 17) (15). In 10 patients, PET/CT revealed carotid arteries FDG uptake considered as active disease. Using the PET/CT as a reference, the sensitivity and specificity of carotid CEUS were 100 and 92%, respectively. Inflammation revealed by PET/CT and neovascularization of the arterial wall revealed by CEUS were correlated (15).

Thus, up to date, the biggest challenge in CDUS as in CEUS is the lack of quantitative, reliable, and effective measures to evaluate disease activity in GCA and monitoring of treatment response.

The objective of this study was to investigate the reliability (consistency and reproducibility) of arterial wall CEUS in GCA with semi-quantitative visual analysis by comparing the classification of different experts on sets of ultrasound loops (inter-rater association) and on experts’ own repeated ratings (intra-rater association).

## Materials and methods

### Inclusion criteria

Inclusion criteria were GCA patients, with American College of Rheumatology (ACR) criteria (19), or age >50 years and CRP >10 mg/L and vasculitis on imaging: ultrasound, MRI, CT or PET/CT (20–23). This study included patients with large-vessel involvement with increased IMT ≥0.8 mm at the axillary or common carotid arteries on CDUS. Patients were included from December 2019 to February 2021.

### Contrast-enhanced ultrasound examination

Contrast-enhanced ultrasound was performed on a Toshiba Aplio 400 ultrasound machine (Canon Medical Systems, Europe) with a L11-4 linear array probe according to the European Federation of Societies for Ultrasound in Medicine and Biology (EFSUMB) guidelines (24). The common carotid artery and axillary arteries were assessed by the same experienced physicians. The ultrasound examination was performed with the patient in the supine position. For each patient, the bilateral carotid and axillary arteries were examined and the wall thickness (IMT) of the common carotid artery and axillary arteries were measured.

The mechanical index was between 0.06 and 0.09. The instrument parameters were kept consistent for all patients. The gray scale was automatically adapted. The maximum IMT was measured using CDUS, and the most prominently thickened vessel segment was chosen based on the accessibility to all parts of the vessel wall. CEUS was performed at the thickest site of the common carotid or axillary artery. Micro Flow Imaging (MFI) mode was used for recording image loops.

Each contrast agent infusion was followed by a saline flush with 10 ml of NaCl 0.9% solution. After injection of 2.5 mL of ultrasound contrast agent (SonoVue, Bracco S.p.A., Milan, Italy), a continuous ultrasound video was recorded over 60 s, and the image loops were stored on an ultrasound machine. Afterward, the recorded movies were analyzed by a real time examination.

### Study design

**FIGURE 1:**
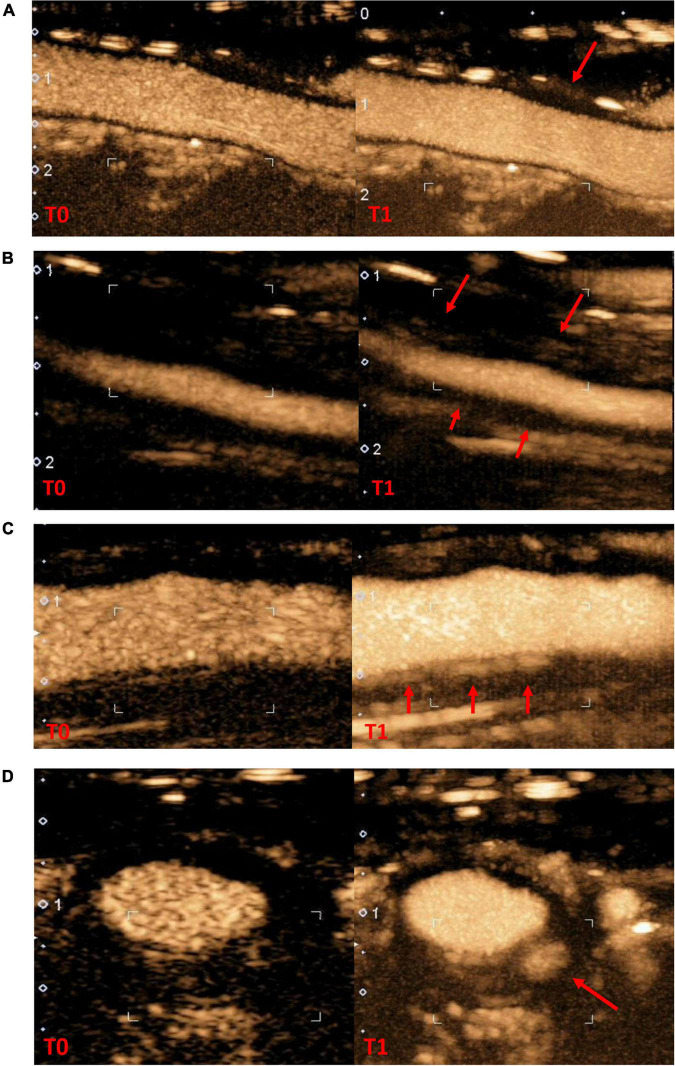
Contrast-enhanced ultrasonography in arterial wall in giant cells arteritis patients **(A),** no contrast-enhanced **(B)**, grade 1 moderate contrast-enhanced **(C)**, grade 2 high contrast-enhanced in longitudinal section **(D)**, grade 2 contrast-enhanced in cross section (T0: wall reference at the start of contrast infusion; T1: wall contrast at the end of the recording, red arrow specific arterial wall thickening area where contrast-enhanced was evaluated).

Images were analyzed by experienced seven vascular physicians (named thereafter observers) from three university hospitals and one general hospital. Consensus meetings were held, a first meeting to specify the evaluation method and comparing the analysis data obtained by the evaluation of three experienced investigators, then each observer analyzed 10 loops of training images and after analysis of the data a second meeting to adjust and harmonize the evaluations was held; then each operator analyzed 4 series of 30 image loops. An initial three-class analysis was performed. The degree of neovascularization at the thickening wall on CEUS was defined as follows (Figure 1): grade 0, no vascularization, representing no moving microbubbles in the thickened artery lesions; grade 1, limited or moderate vascularization, representing limited or moderate visible appearance of microbubbles in the thickened artery lesions; and grade 2, severe vascularization, representing extensive wall vascularization with a clear visible appearance of microbubbles (Supplementary Video 1) (14). A second two-class analysis was then performed, describing no wall contrast or arterial wall contrast. The order of reviewing the image sets was different between each series; the review of each image set was performed with at least a 2-week interval to reduce recall bias. The investigators were blinded to clinical and biological data.

### Ethics

This study was conducted in compliance with the Declaration of Helsinki principles and received ethics approval by the local ethics committee of the University Hospital of Nantes. Each patient included in this study received written information and no patient objected to this study. No written informed consent was needed by the ethics committee because of the retrospective study design (French public health code article: L 1121-1).

### Statistical analysis

Agreement and association measures are used to quantify the degree of consistency between experts’ categorical (e.g., binary or ordinal) ratings. For ordered ratings, measures of association are recommended since diminishing credit is assigned for pairs of ratings on the same patient’s test result which are similar but not in full agreement. Measures of agreement are focusing on assessing the levels of exact concordance (i.e., where raters assign the exact same category to a subject’s test result), whereas measures of association also take into account the degrees of disagreement among raters’ classifications.

In this design, a group of raters scored a set of patients’ test results twice, leading to dependencies between classifications. Hence we applied the modeled based kappa evaluation developed by Nelson et al. that provides an overall evaluation (consistency and reproducibility) of the association among multiple raters’ paired scores of patients’ imaging results, at two points of time (25, 26). As no intervention was planned between the two points of time, the consistency between rater’s paired assessments is determined by the intra-rater association whereas the reproducibility is at tested by the inter-rater association.

Statistical analysis Inter- and intra-observer agreements (or association) were interpreted by the Landis and Koch interpretation: 0.21–0.40: fair; 0.41–0.60: moderate, 0.61–0.80: substantial; ≥0.81: almost perfect.

## Results

We included 30 patients with newly diagnosed or known GCA with LVV of the axillary and/or carotid arteries. The mean age of all included patients was 75.7 ± 5.7 years. The patients were predominantly female: 63.3% (19 females). The mean wall thickness was 2.1 ± 1.1 mm.

For the whole of the observers, the mean number of views of each ultrasound loop was 4.4 ± 1.1 in three-class scale analyses. Inter and intra−observer agreements of arterial wall contrast-enhanced ultrasonography in three-class analysis are summarized in Table 1. The intra-observer association is high (0.7), indicating substantial consistency between both evaluation series at time 1 and time 2 by each observer. The inter-observer association at time 1 is low (0.27) and at time 2 is even lower (0.22). This indicates that consistency between raters is no more than fair.

**TABLE 1:** Inter and intra-observer agreement of arterial wall contrast-enhanced ultrasonography in three-class scale analysis.

Measure of association	Estimated kappa	(95% CI)
Intra-rater	0.70	(0.65; 0.75)
Inter-rater (1st evaluation)	0.27	(0.19; 0.35)
Inter-rater (2nd evaluation)	0.22	(0.15; 0.29)

The agreement of arterial wall contrast in two classes is presented in Table 2. The two-class assessment modestly increases inter-observer agreements, moving from fair to moderate agreement. However, the intra-rater agreement is lower than in the three-class evaluation, indicating a lesser consistency in the evaluation between two views of ultrasound loops.

**TABLE 2:** Inter-observer agreement of arterial wall contrast-enhanced ultrasonography in two-class scale analysis (CI: *confidence interval*).

Measure of agreement	Estimated kappa	(95% CI)
Intra-rater	0.56	(0.43; 0.69)
Inter-rater (1st evaluation)	0.35	(0.21; 0.57)
Inter-rater (2nd evaluation)	0.41	(0.25; 0.60)

Out of the 30 image loops, 13 had an arterial wall thickness greater than 2 mm. Inter and intra−observer agreement of arterial wall contrast-enhanced ultrasonography in three-class or two-class analysis, according to an arterial wall thickness less or greater than or equal to 2 mm are presented in Tables 3,4. In the cases, the intra-observer association ranges from 0.53 to 0.69, indicating moderate to substantial consistency between both evaluation series by each observer. The inter-observer agreements were fair ranging from 0.25 to 0.35 in the three-class analysis. In the two-class analysis, they showed a great variability especially for walls <2 mm.

**TABLE 3:** Inter and intra-observer agreement of arterial wall contrast-enhanced ultrasonography in three-class scale analysis according to an arterial wall thickness (CI: *confidence interval*).

Measure of association	Estimated kappa	(95% CI)
**Arterial wall thickness <2 mm**		
Intra-rater	0.69	(0.59; 0.79)
Inter-rater (1st evaluation)	0.35	(0.20; 0.50)
Inter-rater (2nd evaluation)	0.35	(0.20; 0.50)
**Arterial wall thickness ≥2 mm**		
Intra-rater	0.53	(0.46; 0.60)
Inter-rater (1st evaluation)	0.25	(0.09; 0.41)
Inter-rater (2nd evaluation)	0.25	(0.09; 0.41)

**TABLE 4:** Inter and intra-observer agreement of arterial wall contrast-enhanced ultrasonography in two-class scale analysis according to an arterial wall thickness (CI: *confidence interval*).

Measure of association	Estimated kappa	(95% CI)
**Arterial wall thickness <2 mm**		
Intra-rater	0.56	(0.42; 0.70)
Inter-rater (1st evaluation)	0.36	(0.19; 0.66)
Inter-rater (2nd evaluation)	0.02	(−0.09; 0.27)
**Arterial wall thickness ≥2 mm**		
Intra-rater	0.57	(0.45; 0.68)
Inter-rater (1st evaluation)	0.22	(0.05; 0.44)
Inter-rater (2nd evaluation)	0.63	(0.59; 0.86)

The physician’s experience did not affect inter- and intra-observer agreements (Tables 5,6). However, the CEUS is globally little performed in GCA, thus none of the physicians has performed more than 300 CEUS in GCA to evaluate disease activity in arterial wall.

**TABLE 5:** Inter and intra-observer agreement of arterial wall contrast-enhanced ultrasonography in three-class scale analysis according to physician’s experience (CI: *confidence interval*).

Measure of association	Estimated kappa	(95% CI)
**Physician with more than 5 years of experience**		
Intra-rater	0.68	(0.66; 0.70)
Inter-rater (1st evaluation)	0.35	(0.32; 0.38)
Inter-rater (2nd evaluation)	0.36	(0.33; 0.39)
**Physician with less than 5 years of experience**		
Intra-rater	0.72	(0.70; 0.73)
Inter-rater (1st evaluation)	0.39	(0.36; 0.42)
Inter-rater (2nd evaluation)	0.38	(0.35; 0.41)

**TABLE 6:** Inter and intra-observer agreement of arterial wall contrast-enhanced ultrasonography in two-class scale analysis according to physician’s experience (CI: *confidence interval*).

Measure of association	Estimated kappa	(95% CI)
**Physician with more than 5 years of experience**		
Intra-rater	0.51	(0.38; 0.65)
Inter-rater (1st evaluation)	0.07	NA
Inter-rater (2nd evaluation)	0.42	NA
**Physician with less than 5 years of experience**		
Intra-rater	0.62	(0.47; 0.76)
Inter-rater (1st evaluation)	0.68	NA
Inter-rater (2nd evaluation)	0.55	NA

## Discussion

This multicenter study is the first to investigate inter- and intra-observer agreements of arterial wall contrast in GCA with visual assessment of contrast. In this study, intra-observer agreement in the analysis of arterial parietal contrast uptake in GCA was good with an analysis performed in three-class scale. On the other hand, the inter-observer agreement is fair with κ between 0.22 and 0.27 for an analysis in three-class scale, the inter-observer agreement is slightly better from fair to moderate when the analysis is performed in 2-class scale with κ between 0.35 and 0.41.

Parietal thickening appears to be important to consider in the visual analysis of contrast uptake since inter-observer agreement in the two-category analysis showed higher agreement rates when the wall had a thickness of ≥2 mm. Thus, it is possible that the performance of semi-quantitative contrast score analysis is different in GCA compared with TA because wall thickenings in TA are often much greater than in GCA (12–15). The thicker the wall the more concordant the assessment between observers, however, it remains insufficiently reproducible in this study to take a decision for therapeutic modification.

The results of this study discuss the value of visual assessment of wall contrast uptake in view of the poor inter-observer agreement. Thus, a study of contrast uptake by quantitative methods seems more interesting during LVV. Some authors have proposed other ways of analyzing contrast intake. As such, Bergner et al. used the difference in contrast-enhanced areas between lumen contrast and arterial wall contrast for the study of contrast uptake (16). To better analyze the arterial wall in the LVV, an automated contrast analysis method with digital detection tools should be validated. For CEUS, Giordana et al. reported a lowering of the gray scale median of the common carotid wall under steroid treatment in TA (27). If CEUS interpretation is efficient and reproducible, it could be used in routine clinical practice; it will make monitoring much easier to repeat, safer, faster, and much more cost-effective than MRI or PET/CT. Thus, CEUS could be a good method to monitor GCA activity with large-vessels involvement. The results highlight the need to increase the reproducibility of CEUS as the inter-observer agreement was disappointing. If these results are confirmed, visual interpretation of CEUS cannot be recommended for LVV evaluation in routine practice. It does not seem appropriate to decide on a treatment change based on a visual analysis of the CEUS.

The variability of ultrasound should be put into perspective with inter-observer agreement variabilities for other imaging techniques. To our knowledge, there is no study that has investigated the concordance between observers for CT in GCA. For PET/CT, visual grading system analysis in four classes with liver uptake as reference had good inter observer agreement with κ from 0.79 to 0.96 (28). In CDUS, the main inter-observer agreement data were performed on the temporal arteries and axillary arteries. For the diagnosis of temporal arteritis, “halo” and “compression” signs were the main CDUS patterns for GCA diagnosis. For the halo sign, the agreement between the observers evaluated on images was 0.95 and the agreement of the halo sign on video loop was 0.84 (9). Compression sign for the diagnosis of temporal arteritis had an excellent inter-observer κ:0.83–0.92 (9, 29). For chronic wall modifications of axillary arteries in GCA, the CDUS inter-reader reliability was κ 0.79–0.80 for and κ was 0.88 for intra-reader agreement (30).

The strengths of this study are the multicenter image analysis, image loops were performed in a single center with an identical image acquisition protocol, a blinded analysis of clinical biological data and imaging such as PET/CT. The limitations of this study include the small number of patients and the absence of a probe motion reduction system to limit motion artifacts that alter the interpretation of image loops with the MFI mode. Concerning the experience acquisition of the physician, CEUS is mainly performed in a few expert centers, unlike GCA diagnostic or follow-up CDUS, because few patients with artery wall thickenings are eligible for CEUS and very few centers realize CEUS. Acquiring experience in performing and interpreting the CEUS seems to us to be more difficult to obtain than mastering the compression sign or the halo sign with the CDUS. Thus, the development of software to assist interpretation seems fundamental to have a better reproducibility of results and to have a quantitative evaluation of the contrast uptake.

## Conclusion

This multicenter study showed that the intra-observer agreement for CEUS was good for the semi-quantitative visual analysis. In contrast, inter-observer agreement was poor for semi-quantitative visual analysis and moderately improved when contrast uptake analysis was binary. A significant parietal thickening improved inter-observer agreement in binary analysis. Prospective studies with digital and automated CEUS analysis should be performed to clarify the interest of CEUS in the follow-up of GCA.

## Data availability statement

The raw data supporting the conclusions of this article will be made available by the authors, without undue reservation.

## Ethics statement

The studies involving human participants were reviewed and approved by the Groupe Nantais d’Ethique dans le Domaine de la Santé. Written informed consent for participation was not required for this study in accordance with the National Legislation and the institutional requirements.

## Author contributions

OE, OR, and F-XL: data collection analysis and interpretation, and drafting of the manuscript. JH, OR, F-XL, AT, AR, GG, and OE: data collection and critical review. OE, OR, and M-AV: methodology, analysis, and interpretation of data. OE: study concept and design, data collection, analysis and interpretation, drafting of the manuscript, and supervision. All authors contributed to the article and approved the submitted version.

## Supplementary material

Supplementary Video 1 Grade 2 contrast-enhanced registration loop of an axillary artery in longitudinal section; left window with grade 2 contrast enhancement with significant thickening of the arterial wall on the right side of the image. On the right window, B-mode video of the same artery, registration was performed automatically at the same time.
